# Update of the quality assurance (QA) results of halothane and caffeine analysis of all current participating malignant hyperthermia units

**DOI:** 10.1186/1471-2253-14-S1-A5

**Published:** 2014-08-18

**Authors:** Nickla Fisher, Helena Wright, Philip Hopkins

**Affiliations:** 1 Leeds Institute of Molecular Medicine, St James's University Hospital, LS9 7TF Leeds, UK

## Background

The provision of a Quality Assurance Scheme for members of the European Malignant Hyperthermia Group (EMHG) is to ensure that the concentrations of halothane and caffeine in the tissue bath are as stipulated in the in vitro contracture test (IVCT) protocol. Confirmation of the correct concentrations in the tissue bath is an essential part of the IVCT protocol.

The focus of this presentation will be to provide an update of the QA results from the analysis of both halothane and caffeine from all of the participating units over a period of three years.

The aims are to show

1. How many malignant hyperthermia (MH) units have achieved the target concentrations of halothane for nominal vaporiser settings of 0.5% (0.11mM) and 2.0% (0.44mM) and caffeine concentrations of 0.5mM and 2.0mM?

2. How these halothane concentrations can vary within each lab?

## Results

Each lab is aware of its own results. Anonymised results from January 2012 to January 2014 will be presented from each participating MH unit.

These will include halothane results for vaporiser settings of 0.5% and 2.0%. The tissue bath concentration is usually lower than the required concentration, but halothane concentrations can also be found to be higher. Halothane concentrations can also vary within each lab.

Caffeine analysis results collected over the past 24 months will also be presented from each participating lab.

